# Supplementary figures and images for: Cohesin SMC1β promotes closed chromatin and controls TERRA expression at spermatocyte telomeres
Source: Life Sci Alliance. 2023 May 9;6(7):e202201798. doi: 10.26508/lsa.202201798 (PMC10172765; doi:10.26508/lsa.202201798)

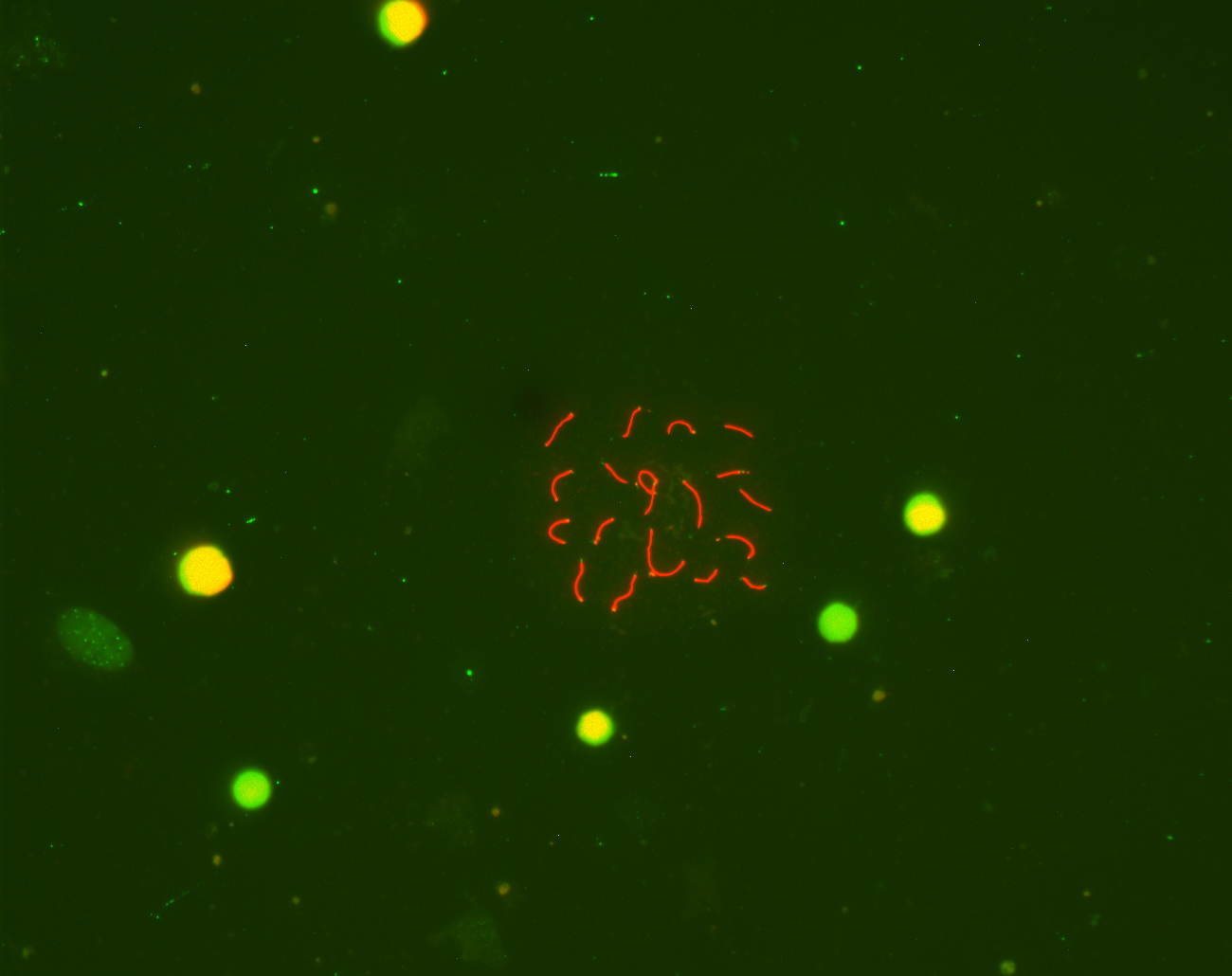

Supplement: Supplementary file 4 [file LSA-2022-01798_SdataF2.zip › Figure 2/Fig 2A/1b-:-1a(c2+c3).TIF]

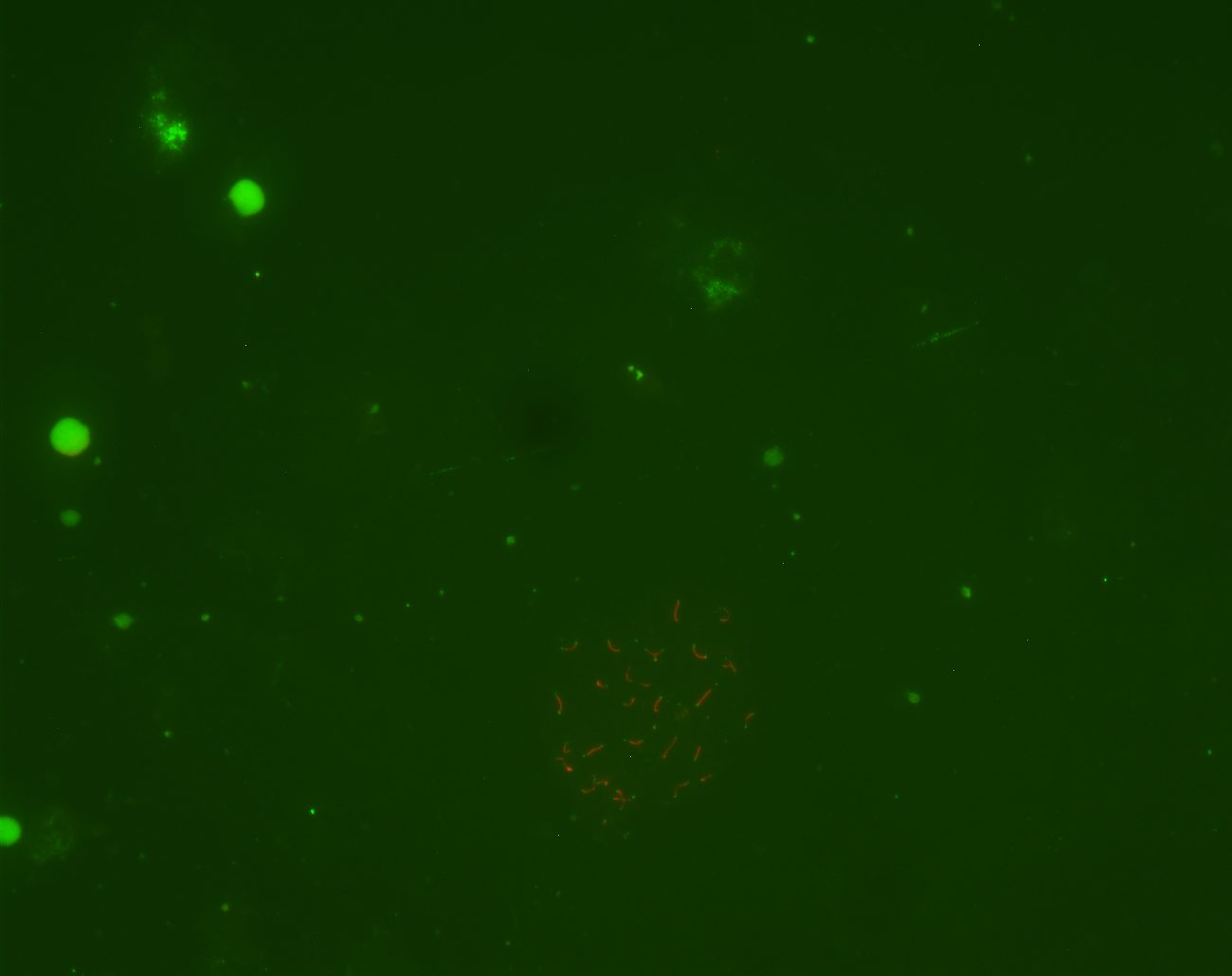

Supplement: Supplementary file 4 [file LSA-2022-01798_SdataF2.zip › Figure 2/Fig 2A/1b -:-(c2+c3).TIF]

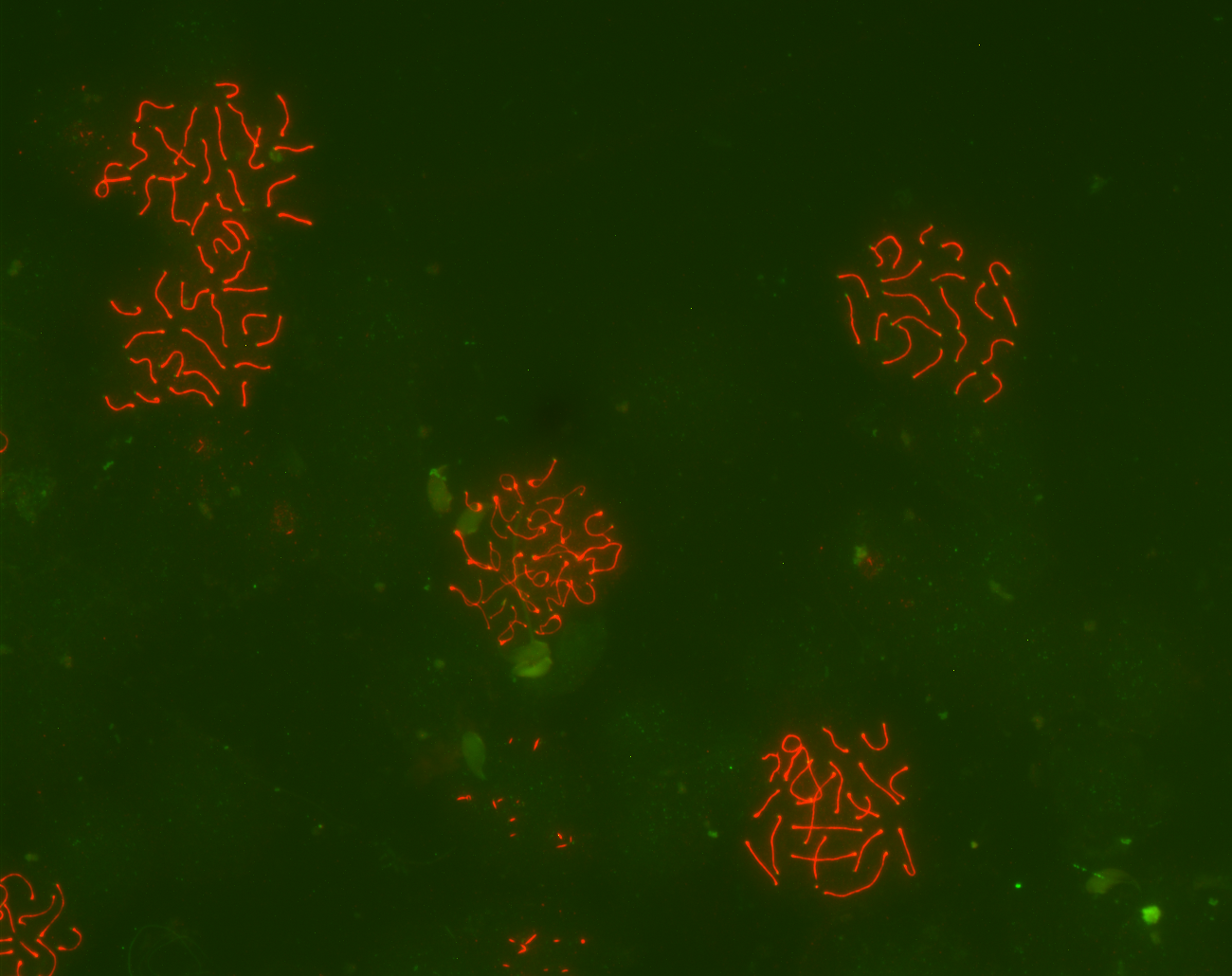

Supplement: Supplementary file 4 [file LSA-2022-01798_SdataF2.zip › Figure 2/Fig 2A/1b+:+ uncropped(c2+c3).TIF]

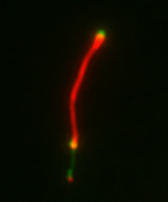

Supplement: Supplementary file 5 [file LSA-2022-01798_SdataF3.zip › Figure 3/Figure 3A/Smc1bko1a DNA TERRA FISH croped image.tif]

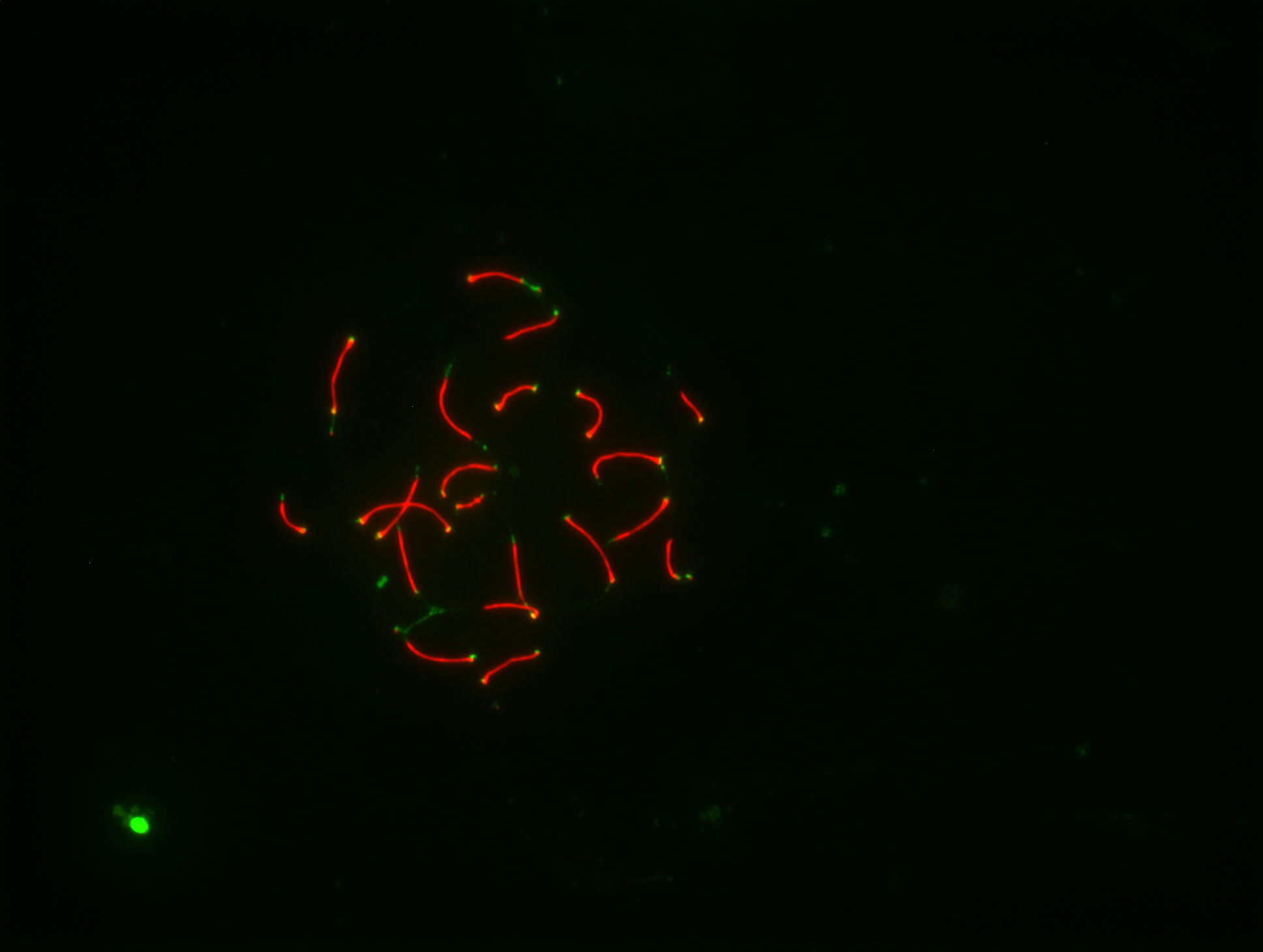

Supplement: Supplementary file 5 [file LSA-2022-01798_SdataF3.zip › Figure 3/Figure 3A/Smc1bko1a DNA TERRA FISH.tif]

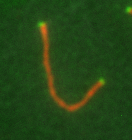

Supplement: Supplementary file 6 [file LSA-2022-01798_SdataFS3.zip › Figure 3 Supplement/Suppl Fig. 3A/WT DNA TERRA FISH croped image.tif]

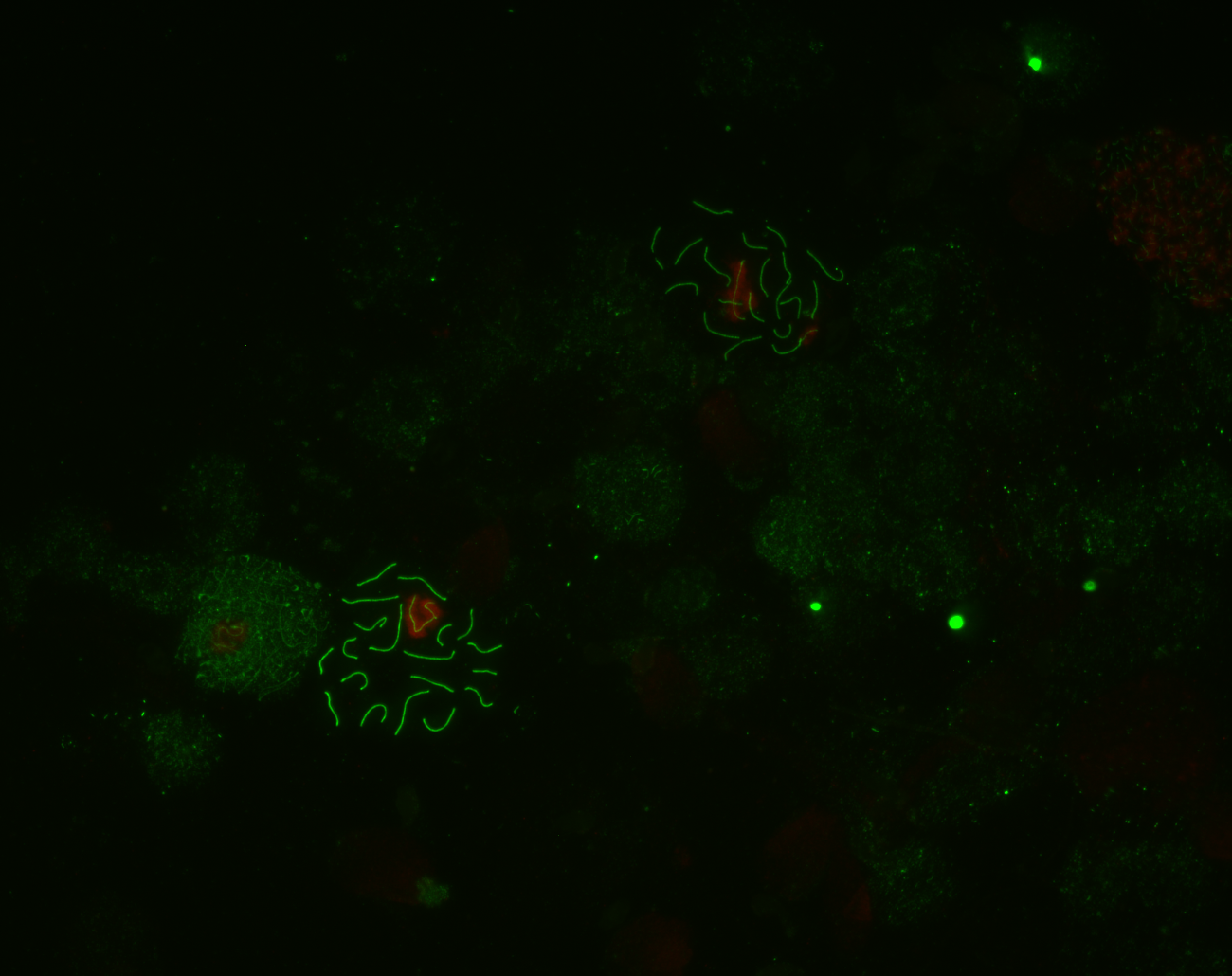

Supplement: Supplementary file 7 [file LSA-2022-01798_SdataF4.zip › Figure 4/Fig 4A/1b+:+ t only(c2+c3).TIF]

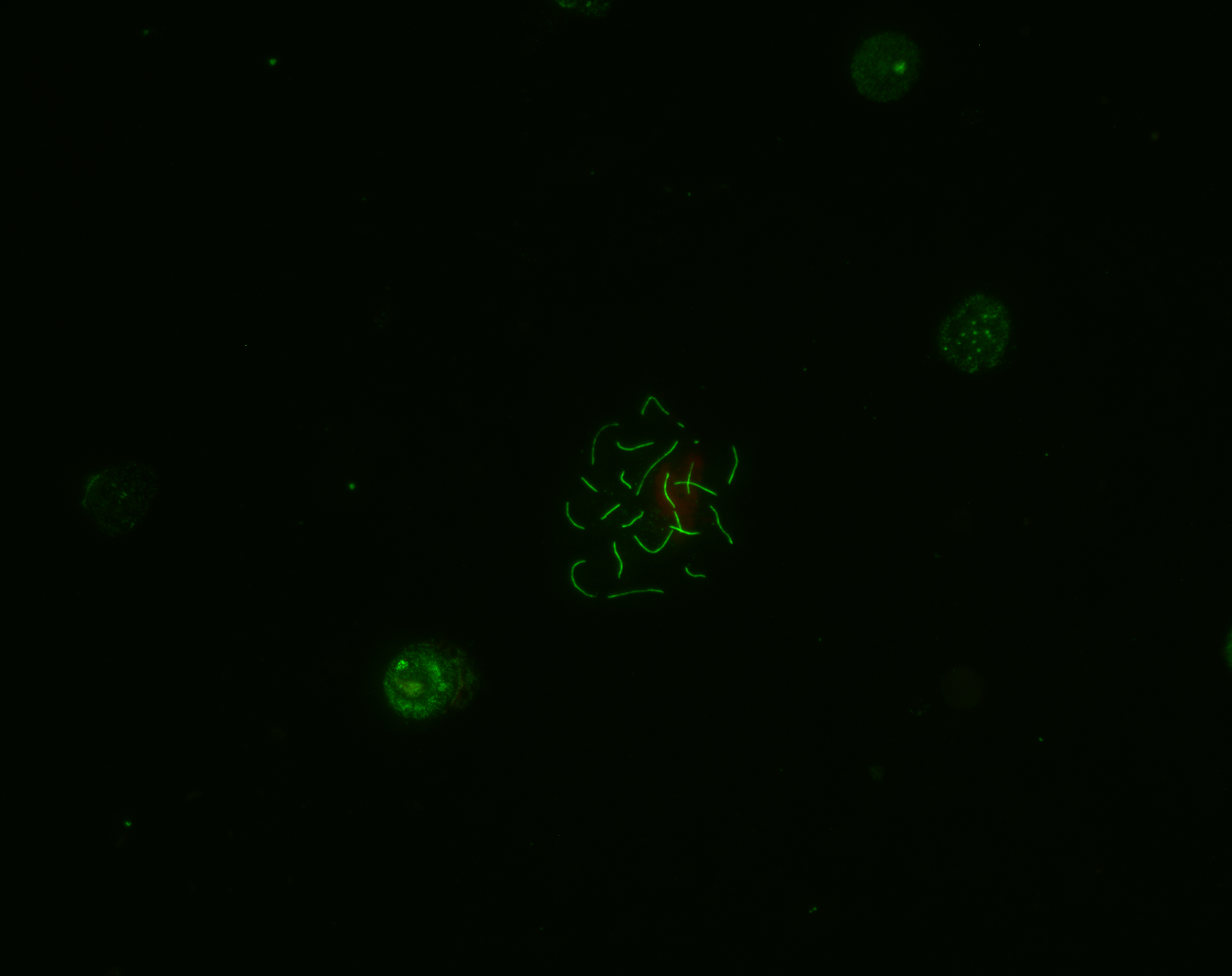

Supplement: Supplementary file 7 [file LSA-2022-01798_SdataF4.zip › Figure 4/Fig 4A/1b-:- 1a h treated.TIF]

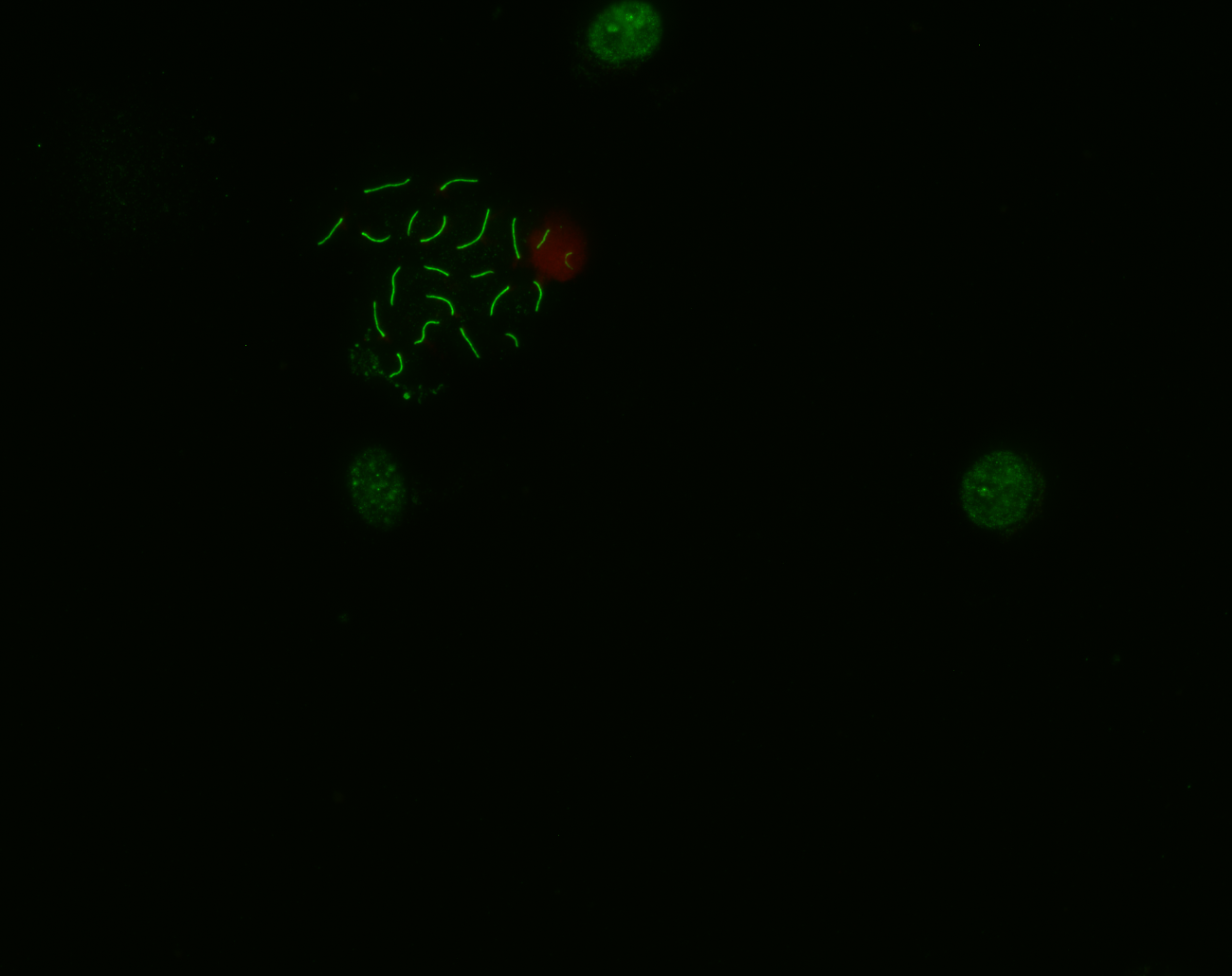

Supplement: Supplementary file 7 [file LSA-2022-01798_SdataF4.zip › Figure 4/Fig 4A/1b-:- 1a untreated(c2+c3).TIF]

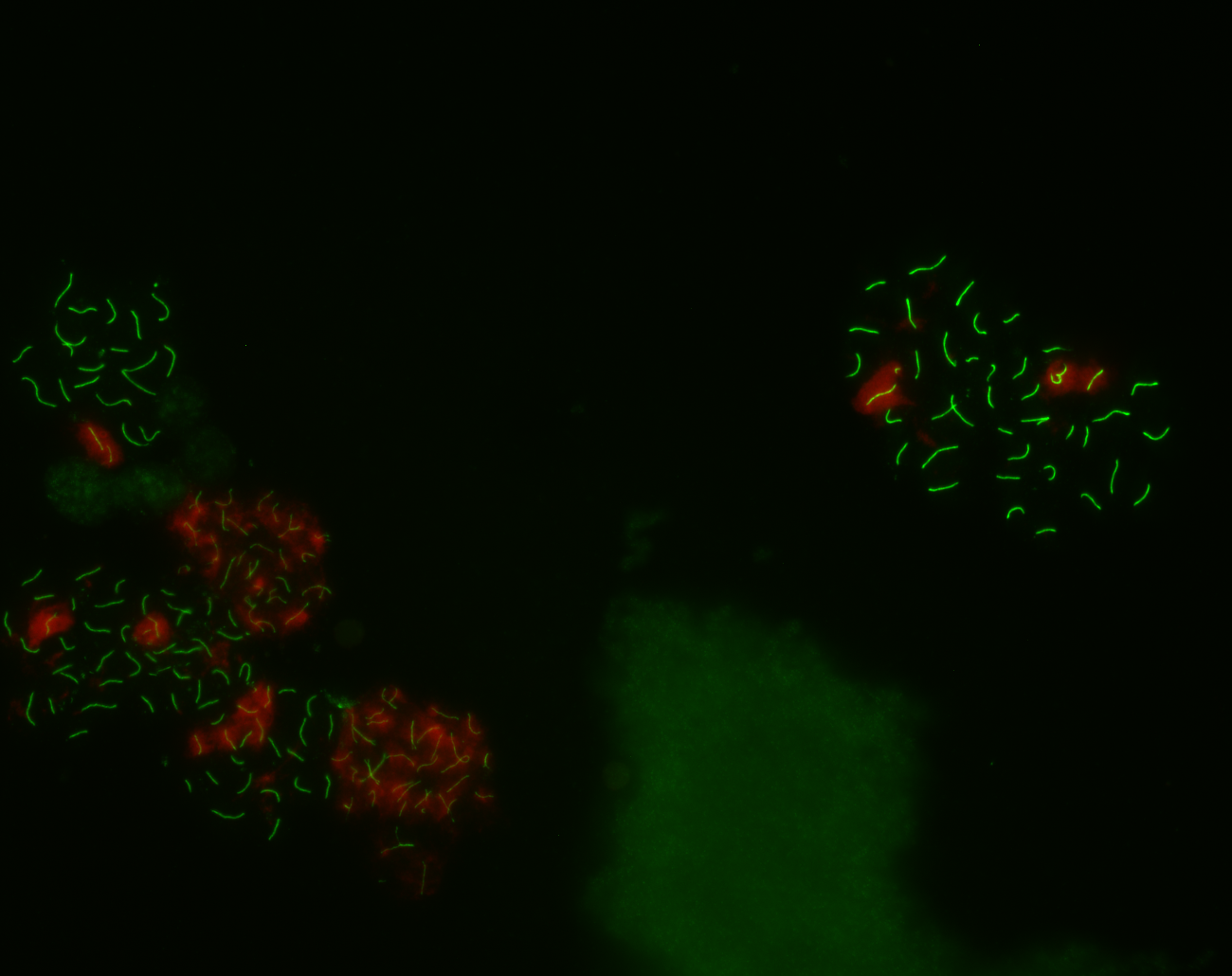

Supplement: Supplementary file 7 [file LSA-2022-01798_SdataF4.zip › Figure 4/Fig 4A/1b-:- 1a h+t treated(c2+c3).TIF]

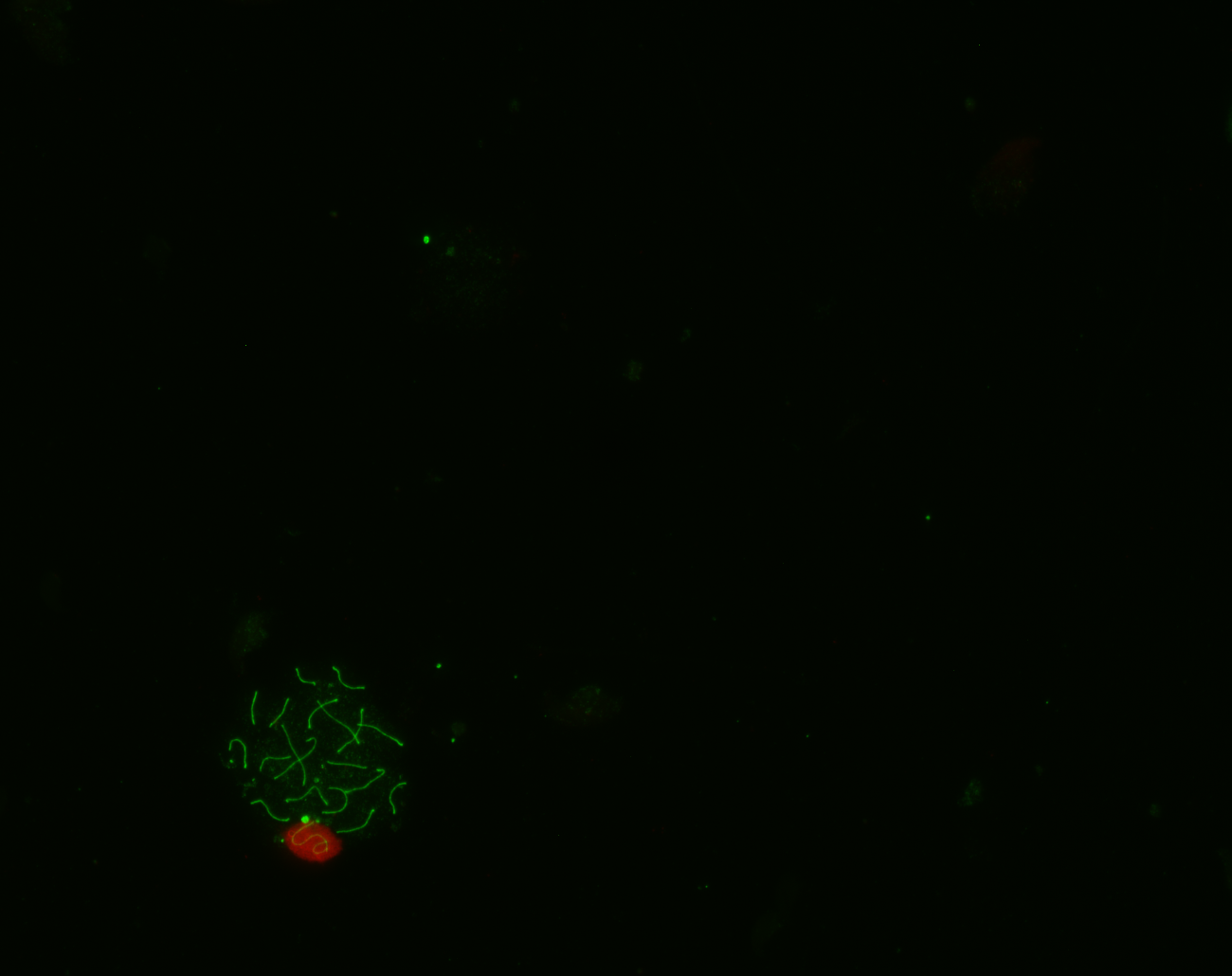

Supplement: Supplementary file 7 [file LSA-2022-01798_SdataF4.zip › Figure 4/Fig 4A/1b+:+ untreated.TIF]

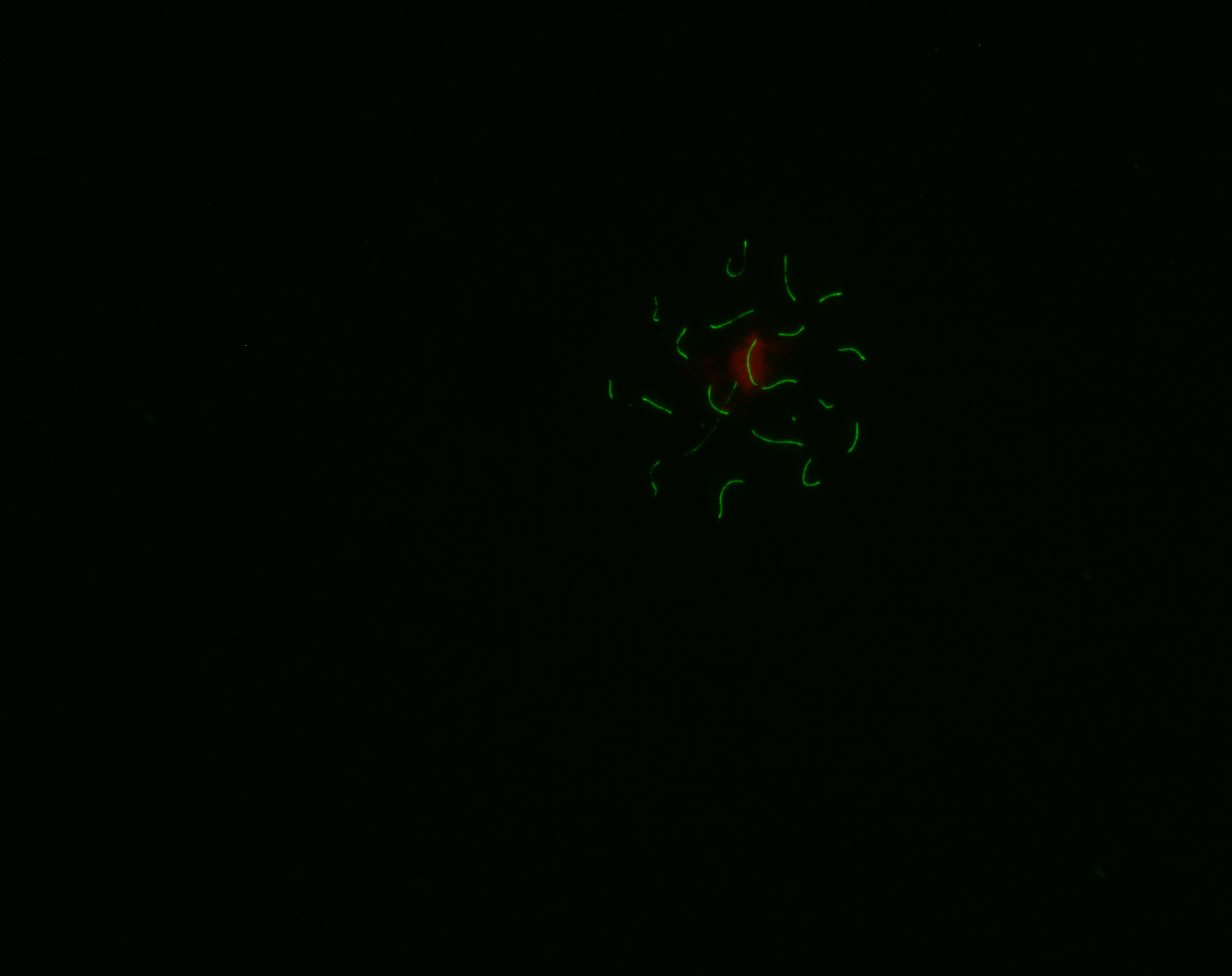

Supplement: Supplementary file 7 [file LSA-2022-01798_SdataF4.zip › Figure 4/Fig 4A/1b-:- 1a t1 only(c2+c3).TIF]

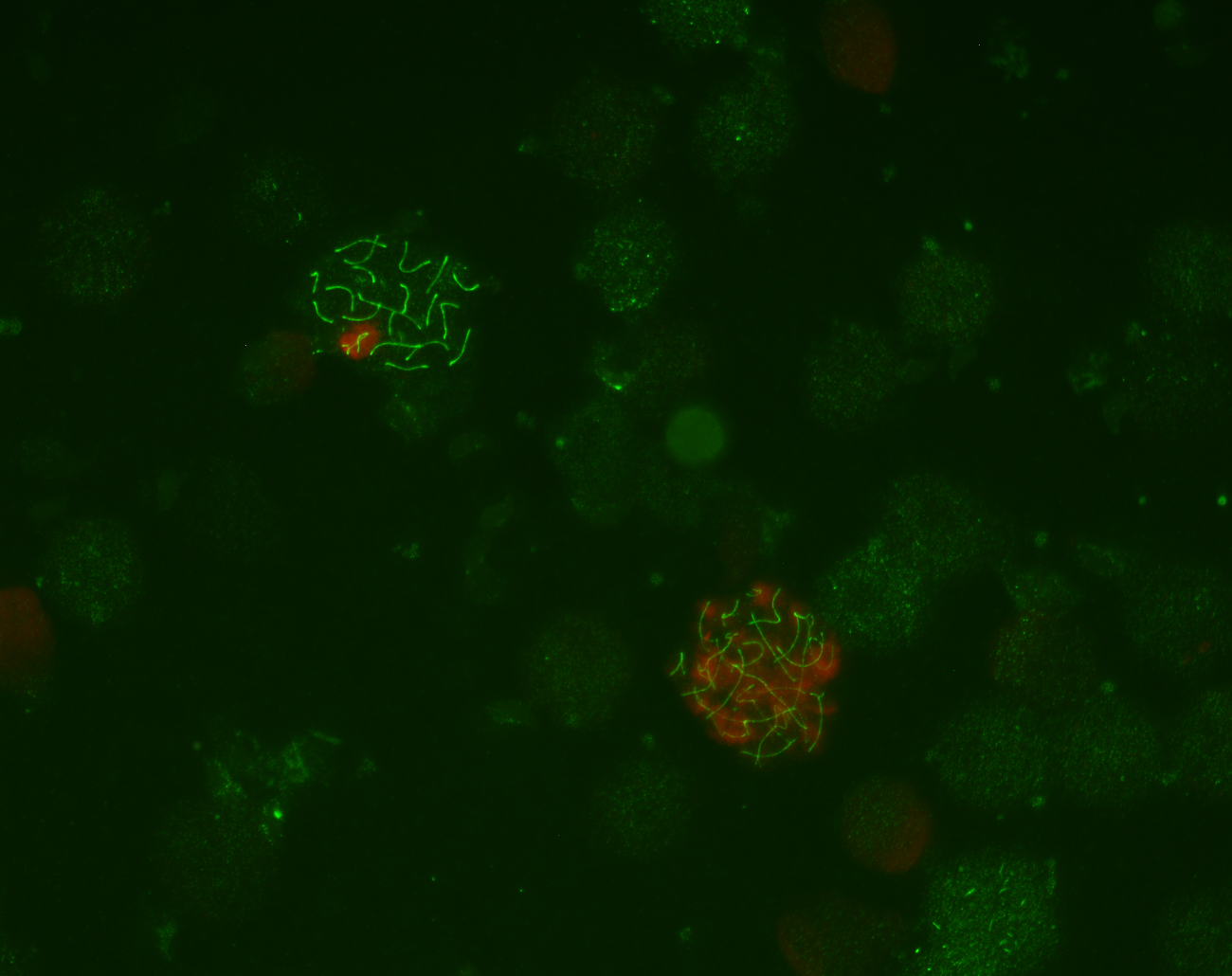

Supplement: Supplementary file 7 [file LSA-2022-01798_SdataF4.zip › Figure 4/Fig 4A/1b+:+ h+t treated(c2+c3).TIF]

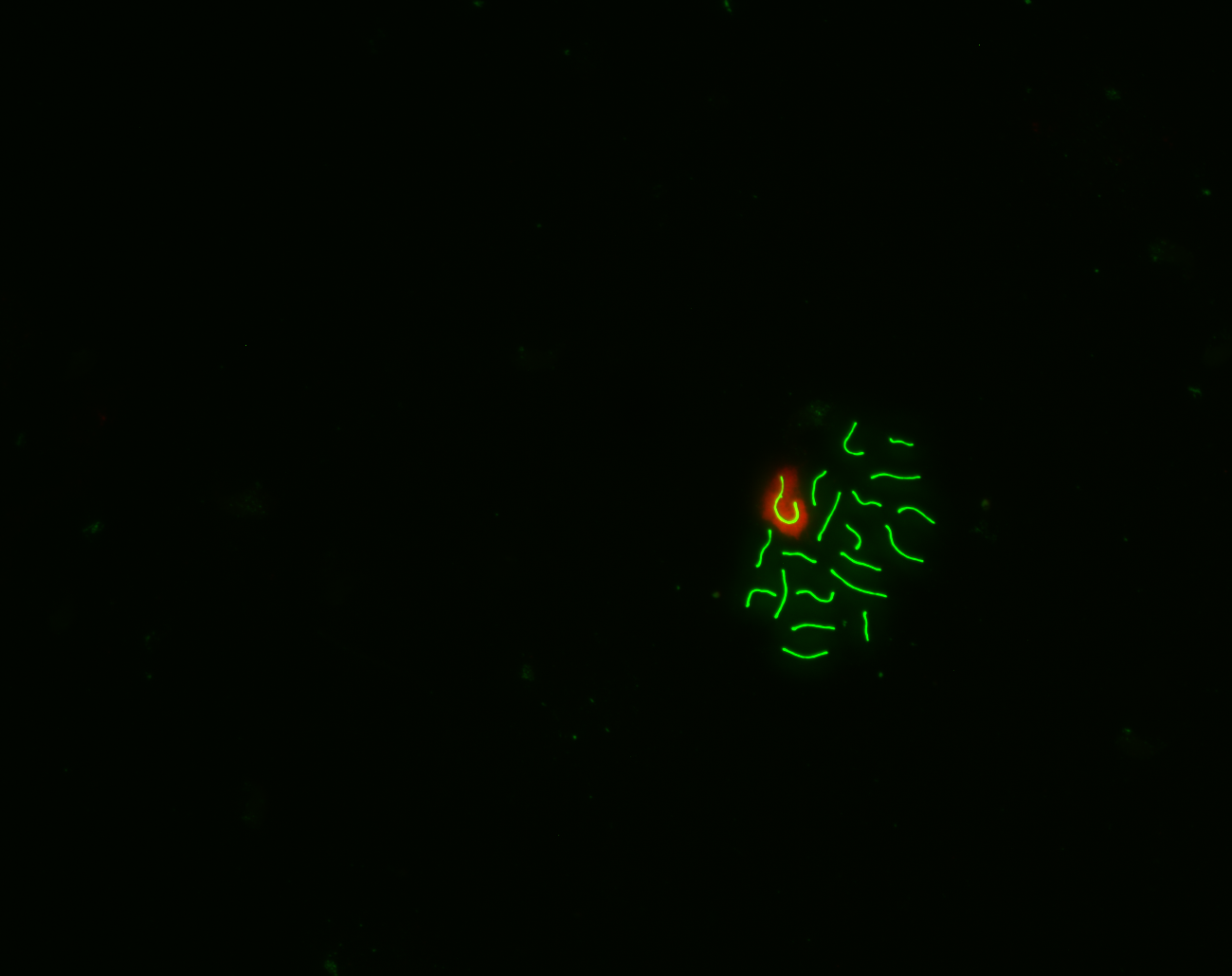

Supplement: Supplementary file 7 [file LSA-2022-01798_SdataF4.zip › Figure 4/Fig 4A/1b+:+ h treated(c2+c3).TIF]
