# Supplementary material for: Cohesin SMC1β promotes closed chromatin and controls TERRA expression at spermatocyte telomeres
Source: Life Sci Alliance. 2023 May 9;6(7):e202201798. doi: 10.26508/lsa.202201798 (PMC10172765; doi:10.26508/lsa.202201798)
Supplement: Supplementary file 9 [file LSA-2022-01798_TableS1.pdf]

Biswas et al., Supplemental Table 1

	Genotype	Significance	P Value
5 kb			
	wt vs 1b-/-	****	<0.0001
	wt vs 1b-/-1a	***	0.0002
	1b-/- vs 1b-/-1a	ns	0.6686
45 kb			
	wt vs 1b-/-	***	0.0005
	wt vs 1b-/-1a	***	0.0005
	1b-/- vs 1b-/-1a	ns	0.9993
450 kb			
	wt vs 1b-/-	*	0.0289
	wt vs 1b-/-1a	**	0.0020
	1b-/- vs 1b-/-1a	ns	0.5727
4500 kb			
	wt vs 1b-/-	ns	0.7864
	wt vs 1b-/-1a	ns	0.1031
	1b-/- vs 1b-/-1a	ns	0.3306
